# Supplementary material for: Effect of Pretreatment on the Nitrogen Doped Activated Carbon Materials Activity towards Oxygen Reduction Reaction
Source: Materials (Basel). 2023 Aug 31;16(17):6005. doi: 10.3390/ma16176005 (PMC10488859; doi:10.3390/ma16176005)
Supplement: Supplementary file 1 [file materials-16-06005-s001.zip › materials-2427639-supplementary.pdf]

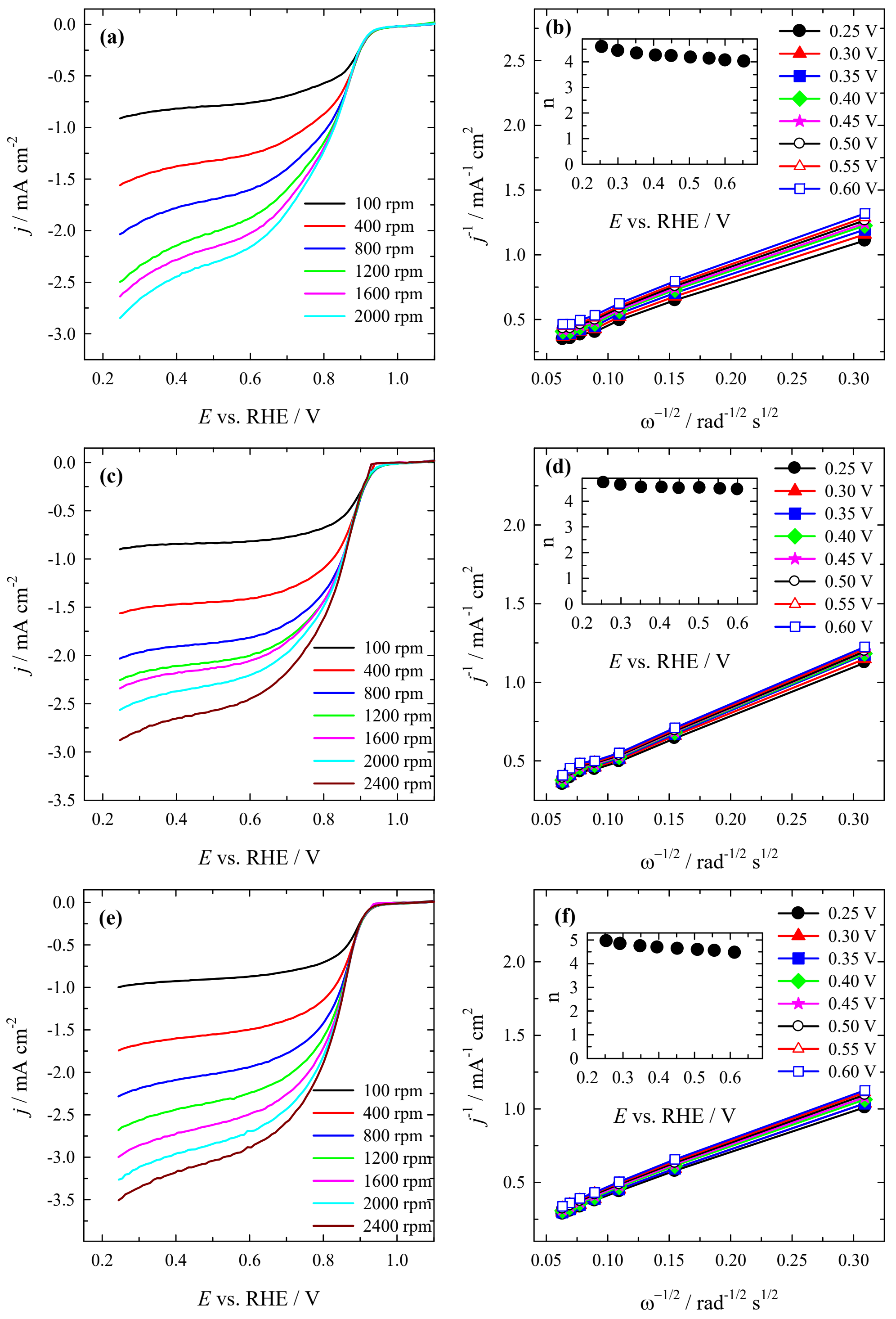

Figure S1. LSVs of APYR-W-N (a), APYR-H-N (c), and APYR-T-N (e) recorded at a scan rate of 10 mV s^{-1} in O_2-saturated 1 M KOH at different rotation rates, (b, d, f) Corresponded Koutecky–Levich plots at electrode potentials of 0.25-0.6 V.

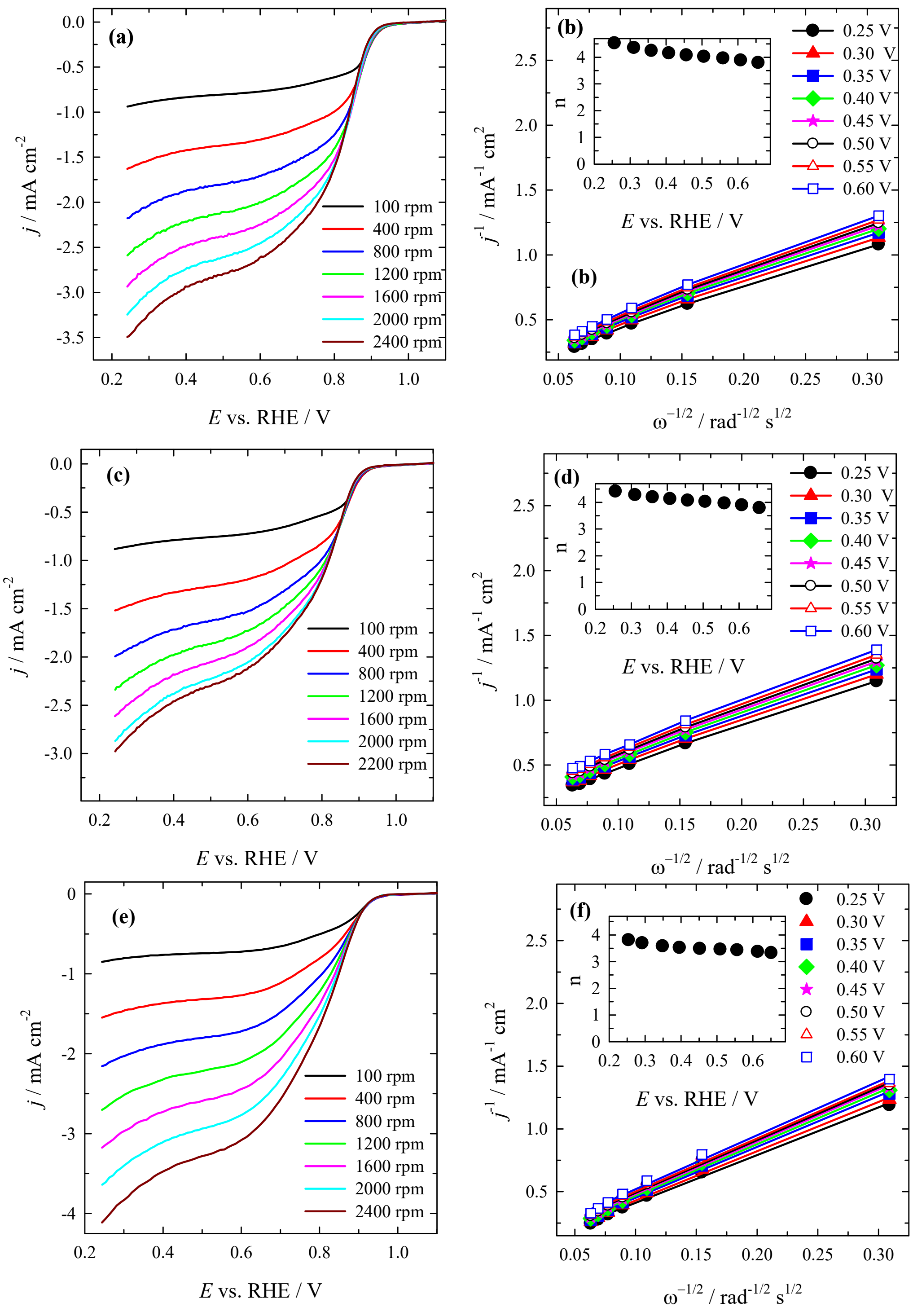

Figure S2. LSVs of AHTC-W-N (a), AHTC-H-N (c), and AHTC-T-N (e) recorded at a scan rate of 10 mV s^{-1} in O_2-saturated 1 M KOH at different rotation rates, (b, d, f) Corresponded Koutecky–Levich plots at electrode potentials of 0.25-0.60 V.

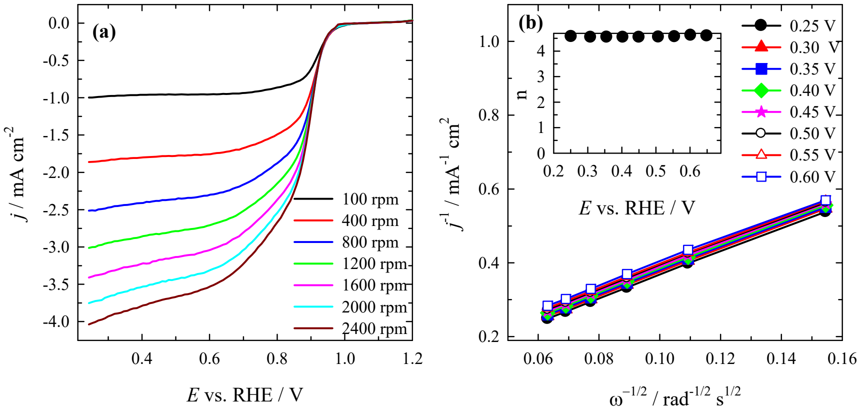

Figure S3. LSVs of Pt/C (a) recorded at a scan rate of 10 mV s^{-1} in O_2-saturated 1 M KOH at different rotation rates, (b) Corresponded Koutecky–Levich plots at electrode potentials of 0.25-0.60 V.
